# Effects of Resistance Exercise on Cerebral Redox Regulation and Cognition: An Interplay Between Muscle and Brain

**DOI:** 10.3390/antiox8110529

**Published:** 2019-11-06

**Authors:** Ricardo A. Pinho, Aderbal S. Aguiar, Zsolt Radák

**Affiliations:** 1 Laboratory of Exercise Biochemistry in Health, Graduate Program in Health Sciences, School of Medicine, Pontifícia Universidade Católica do Paraná, 80215-901 Curitiba, Brazil; 2 Exercise Biology Lab, Department of Health Sciences, Federal University of Santa Catarina, 88.906-072 Araranguá, Brazil; aderbal.aguiar@ufsc.br; 3 Research Institute of Sport Science, University of Physical Education, H-1123 Budapest, Hungary; radak@tf.hu

**Keywords:** resistance training, physical exercise, redox, oxidative stress, BDNF, cognition, brain

## Abstract

This review highlighted resistance training as an important training type for the brain. Most studies that use physical exercise for the prevention or treatment of neurodegenerative diseases have focused on aerobic physical exercise, revealing different behavioral, biochemical, and molecular effects. However, recent studies have shown that resistance training can also significantly contribute to the prevention of neurodegenerative diseases as well as to the maintenance, development, and recovery of brain activities through specific neurochemical adaptations induced by the training. In this scenario we observed the results of several studies published in different journals in the last 20 years, focusing on the effects of resistance training on three main neurological aspects: Neuroprotective mechanisms, oxidative stress, and cognition. Systematic database searches of PubMed, Web of Science, Scopus, and Medline were performed to identify peer-reviewed studies from the 2000s. Combinations of keywords related to brain disease, aerobic/resistance, or strength physical exercise were used. Other variables were not addressed in this review but should be considered for a complete understanding of the effects of training in the brain.

## 1. Introduction

**Figure 1:**
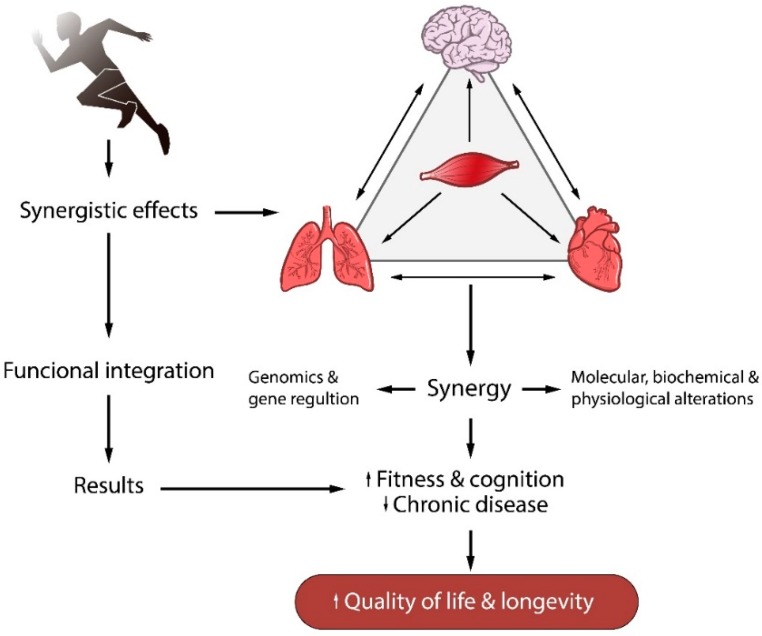
Interactions between bodily systems from regular physical exercise. A synergistic effect between muscle, brain, and heart which modulates molecular, biochemical, and physiological changes, decreasing the risk of chronic diseases.

Most studies suggest the use of physical exercise to decrease the risk of cardiovascular [1], pulmonary [2], immunological [3], metabolic [4], and neurodegenerative diseases [5]. Although the cellular mechanisms that are regulated by physical exercise and that reduce the risk of chronic diseases are not fully understood, it is widely accepted that, overall, regular physical exercise promotes synergistic effects in different organs and tissues, especially between muscle and brain, muscle and lungs, and muscle and heart. Furthermore, this synergy modulates gene expression and causes molecular, biochemical, and physiological changes. These changes promote interactions between bodily systems, improving physical fitness and cognitive performance and, consequently, decreases the risk of chronic diseases (Figure 1).

Despite aerobic exercise being more associated with neuroprotective mechanisms [6], recent studies have shown that resistance training also can significantly contribute to the prevention of neurodegenerative diseases [7,8,9,10] as well as to the maintenance, development, and recovery of brain activities through specific neurochemical adaptations induced by the training [11]. These biological changes induced by resistance training depend on the duration, intensity, frequency, and type of exercise which constitutes the basic parameters of an exercise training program for health [12]. Different from aerobic exercise in the form and intensity of execution and in the recruitment of energetic substrates, resistance training is performed against an external resistance to increase muscular strength and/or mass. This type of training depends primarily on anaerobic metabolism and promotes different stimuli depending on the intensity of muscular contraction, which affects muscle homeostasis. Resistance training induces muscle mechanical tension and increases intracellular calcium concentration. These changes activate different signaling pathways such as extracellular signal regulated kinase ERK/c-Jun N-terminal kinase (JNK), Ca^2+^/calmodulin-dependent protein kinase II (CaMKII), and fodfatidilinositol 3-quinase (PI3K)/protein kinase B (AKT)/ mammalian target of rapamycin (mTOR) which act upon specific targets and modulate gene expression through transcription and translation processes [13]. The effects of resistance exercise on skeletal muscle are well understood but effects on the brain have only been partially elaborated and are not always consistent.

The brain is susceptible to physical exercise by a change in the neuronal redox state. The acute response to physical exercise increases blood flow and enhances cell metabolism [14], adaptive changes include the positive regulation of antioxidants and the repair of enzymes, mitochondrial biogenesis, and redox regulation by different signaling pathways [6]. Such effects of physical training on the brain are derived from studies on aerobic training. However, in recent years, the number of experimental studies related to the effects of resistance (or strength) training on the brain have increased significantly, but the mechanisms are not yet fully understood. The aim of this review was to search recently published literature for studies investigating the effects of resistance training on three main neurological aspects: Neuroprotective mechanisms mediated by brain-derived neurotrophic factor (BDNF), oxidative stress, and cognition.

## 2. Mechanism of Resistance Exercise-Induced Neuroprotection: The Role of BDNF

According to Varendi and colleagues [15], the BDNF gene encoding human BDNF is located on chromosome 11 and eight different promoters result in varied mRNA transcripts. BDNF mRNA contains two alternative polyadenylated transcription stop sites providing binding sites for microRNAs in post-transcriptional regulation. More than 20 microRNAs are thought to control BDNF by its 3′UTR in vitro, but only one, miR-206, has been shown in vivo [15]. The BDNF protein is produced by many brain structures and other tissues such as the retina, motor neurons, kidneys, salivary, and prostate [16]. BDNF is also produced in skeletal muscle and could play an important role in the development of these muscles, as well as in degenerative muscular diseases [17]. After sciatic nerve damage, increases in mRNA and protein levels of BDNF were highly significant in skeletal muscle, suggesting a role of BDNF in repair mechanisms and possibly satellite cell activation [18]. Indeed, the ablation of BDNF in skeletal muscle was associated with a marked decrease in the satellite cell number, suggesting that BDNF could be an important regulator at the early stage of muscle repair via the activation of satellite cells [19].

The effects of exercise on BDNF production could be systemic however, exercise mediated changes in BDNF levels in the central nervous system have been the most studied to date. BNDF acts on certain neurons in the central and peripheral nervous system, regulating existing neurons and stimulating the growth and differentiation of new ones [20]. As one of the main regulators of brain metabolism, BNDF plays the main role in the development of synaptic plasticity and thus has attracted the attention of researchers interested in neurodegenerative diseases. Pre-clinical trials have shown correlations between depressive behaviors and decreased BNDF levels in the hippocampus [21]. Similar results exist regarding Parkinson’s [22] and Alzheimer’s disease [23]. In experimental models, the brain levels of BDNF and other neurotrophins decreased after exposure to toxins such as 1-methyl-4-phenyl-1,2,3,6-tetrahydropyridine (MTPT) and 6-hydroxydopamine (6-OHDA) [8,9,24,25,26,27].

It has been reported that BDNF is involved in neural survival and differentiation [28] which could be closely linked to cellular energy levels. Indeed, it has been shown that BDNF can enhance glucose uptake in response to increased energy needs by stimulating the expression of GLUT3 [29]. Thus, BDNF mediated enhanced metabolism can cover the energy cost of increased amino acid uptake and associated protein synthesis necessary for neuronal differentiation, as well as the branching of axons and dendrites [29,30]. Indeed, energy costs of protein synthesis are significantly higher than the turnover cost of oligonucleotides or lipids [31]. However, the greatest extent of energy expenditure in the brain is consumed by action potential related events [32] and BDNF is also an active regulator of synaptic transmission [33]. Based on this, it is not surprising that the expression of BDNF is under neuronal activity-dependent calcium signaling [34]. Indeed, it is well documented that physical exercise-induced beneficial structural and functional effects are associated with the upregulation of BDNF levels. 

The results of the effect of resistance training on BDNF levels are diverse. Studies in humans showed that no alterations [35,36,37] or increases [38,39,40,41,42,43] were reported in the level of circulating BDNF. One study observed a difference between the sexes and an increase in BDNF levels in males, but not in females, was noted [44]. It has been suggested that the brain is the main source of the increased BDNF level in circulation after endurance exercise [45,46]. However, the source of increased BDNF in the circulation after resistance training is still unknown. Due to the difficulties of resistance training methods in animal models, BDNF studies with resistance training are limited. When Wistar rats were subjected to four weeks of progressive strength exercise in a vertical ladder apparatus, increased neurogenesis was suggested based on Ki-67-positive cells, but no change in BDNF level was reported in the hippocampus [47]. We have investigated the effects of aerobic and strength training on BDNF levels and neuroplasticity, and found that both endurance and resistance training results in similar stimulating effects on BDNF levels in rats [48]. 

## 3. Oxidative Stress in the Brain and Resistance Exercise

The brain is highly sensitive to oxidative stress due to its high levels of phospholipids and polyunsaturated fatty acids [6]. These molecules are prone to oxidation, which leads to the production of abundant reactive oxygen species (ROS) [49]. Moreover, the brain has low levels of antioxidant enzymes and certain regions, like the striatum, have high iron levels that facilitate the formation of ROS [49,50,51,52]. Consequently, the brain’s susceptibility to oxidative stress reduces BDNF levels, since these are influenced by changes in the brain redox state [6,53].

Aerobic physical training has been used in different studies in humans [54,55,56,57] and animals [9,58,59,60,61,62,63,64,65,66] to reduce oxidative stress and maintain the brain redox balance as well as increase the BDNF levels [6]. The influence of aerobic exercise on the brain redox system has been widely reported by different researchers but regarding the cerebral role of resistance exercise, the results are not fully understood. Although several studies have been conducted to verify the effects of exercise on different brain functions and mechanisms, it is only in recent years that the role of resistance exercise has effectively drawn the attention of researchers. For example, in the last 10 years several studies have reported the neuroprotective capacity of resistance exercise, however, few of these considered oxidative stress (see Table 1). 

Although the cellular mechanisms involved in the regulation of brain oxidative stress by resistance exercise are not fully understood, it is possible to speculate that the adaptive changes induced by resistance training from muscle involve the up-regulation of antioxidant and brain redox regulation from different proteins and pathways, such as the mammalian target of rapamycin (mTOR), a serine/threonine kinase important for cell growth, proliferation, and survival of brain [78] as well as the cAMP-response element-binding protein (CREB), an intracellular protein that regulates the expression of genes that are important in dopaminergic neurons [79]. Both mTOR and CREB are responsible for enhanced translation initiation from AKT (protein kinase B) phosphorylation, which leads to both muscle and brain BDNF expression and activation [80,81,82]. 

BDNF release from muscle contraction reaches the brain and binds to Tropomyosin receptor kinase B (TrkB) to induce phosphorylation of different cascades of signaling pathways: PI3K/AKT/mTOR, Pi3K/AKT/CREB, Pi3k/ERK/CREB, and phospholipase Cγ (PLCγ)/CamKII/CREB. The activation of these different signaling pathways results in the additional secretion of the BDNF. In additional, it is possible that the brain mTOR and CREB signaling are also important targets of resistance exercise. These observations are supported partially by previous studies that showed PI3K/mTOR signaling [83] and elevated BDNF levels binds the TRKb receptors and PKC/CREB [48,84] after resistance exercise.

**Figure 2:**
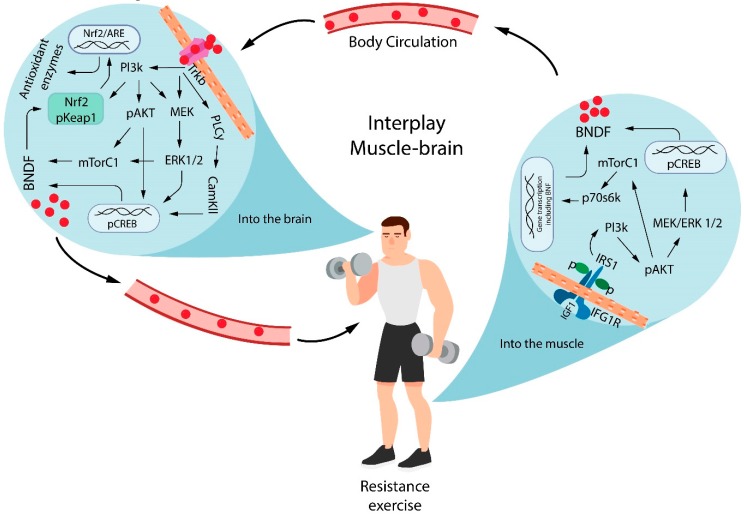
Interplay between muscle and brain in BDNF-mediated redox regulation. Resistance exercise induces BDNF generation from CREB and mTor phosphorylation by the Pi3K/AKT signaling pathway. The BDNF release from muscle contraction reaches the brain and binds the TrkB receptor to induce the phosphorylation of different cascades of signaling pathways, which results in the additional secretion of BDNF. Brain BDNF leads to the activation of Nrf2, which regulates the expression of antioxidants molecules. BDNF =brain-derived neurotrophic factor; IGF1 = insulin-like growth factor 1; IGF1R = insulin-like growth factor 1 receptor; Pi3K = phosphatidylinositol 3-kinase; IRS1 = Insulin receptor substrate 1; pAKT = protein kinase B phosphorylated; MEK = mitogen-activated protein kinase; ERK = extracellular signal–regulated kinase; CREB = cAMP-response element-binding protein phosphorylated; mTORC1 = mammalian target of rapamycin complex 1; p70s6k = ribosomal protein S6 kinase beta-1; TrkB = Tropomyosin receptor kinase B; PLCγ = phospholipase C gamma; CamKII = calcium/calmodulin-dependent protein kinase II; ARE = antioxidant response element; pKeap1 = Kelch-like ECH-associated protein 1 phosphorylated; Nrf2 = nuclear factor erythroid 2-related factor 2.

The BDNF leads to the activation of nuclear factor erythroid 2-related factor 2 (Nrf2) [85], which regulates the expression of detoxification enzymes and antioxidants to protect brain cells from oxidants, electrophiles, and inflammatory agents [86], as well as to maintain the mitochondrial function, cellular redox, and protein homeostasis [87]. Nrf2 is a cellular regulator of antioxidant defense systems [88]. Under physiological conditions, the Nrf2 is linked in the cytoplasm to the Kelch-like ECH-associated protein 1 (keap1). Nrf2 translocates to the nucleus in response to oxidative stress or when electrophilic molecules that covalently modify cysteine residues present in the thiol-rich KEAP1 protein by oxidation or alkylation [89,90] where it binds to specific DNA sites termed anti-oxidant response elements (ARE) to initiate the transcription of cytoprotective genes such as heme oxygenase-1 (HO-1), superoxide dismutase (SOD), glutathione *S*-transferase (GST), NAD(P)H: quinone oxidoreductase 1 (NQO1), and γ-glutamatecysteine ligase (GCL) [91]. This mechanism is summarized in Figure 2.

Previous studies have showed that strength training promotes the upregulation of Nrf2 in the central nervous system after experimental autoimmune encephalomyelitis (EAE) induction [7]. Similar results were previously reported by Aguiar et al. [66], who demonstrated that moderate-intensity physical exercise protected the 6-OHDA-induced loss of tyrosine hydroxylase immunolabeling and activated the Nrf2-ARE pathway in the nigrostriatal pathway. Regulation of antioxidant enzyme activity by resistance exercise has not been the focus of many studies. In one of the few studies, Park et al. [71] showed that resistance exercise training increased SOD1 activity in the hypothalamus of rats with type II diabetes (T2DM) and that it could contribute to hypothalamus redox regulation under T2DM conditions. Souza et al., [7] showed that animals with EAE, undertaking resistance training, showed no changes in SOD activity, but a modulation in the content of glutathione and glutathione peroxidase activity was observed. The authors suggest that these exercise-modulating effects on the glutathione system may be associated with the regulation of mechanisms controlled by Nrf2 phosphorylation. These results suggest the possibility of a regulatory mechanism induced by resistance exercise which modulates the actions of BDNF and provides evidence of resistance training-induced brain redox modulation.

## 4. Resistance Exercise and Cognition 

There is not much evidence regarding the effects of resistance training on higher brain functions such as cognition, executive function, and attention. The primary outcome of major clinical trials was muscle strength [92,93,94], fall prevention [95], and the neuronal effects were secondary outcomes. These clinical studies focused on healthy or unhealthy aged people using various neurological tests such as the Working Memory Index for working memory [92], the Rey Auditory Verbal Learning Test for declarative memory [96,97], Trail Making Test Part B, Verbal Digits Backward Test, and Stroop Color-Word Test for executive functions [95,96,97], as well as the Alzheimer’s Disease Assessment Scale (ADAS-Cog) and Mini-mental State Examination for cognitive impairments [93,94,97]. The most consistent result of resistance training was the improvement of the executive function in the elderly with no differences between the sexes. Three studies using male rats and a vertical ladder where the cognitive enhancer effects of resistance training were associated with neurogenesis [47], an improved IGF-1 pathway in young rats [98], and BDNF/TrκB signaling in aged rats [48].

Cognition is the ability to process information through perception, knowledge acquired through experience and context, and personal characteristics that integrate all information to interact with the environment. Aging and many neurological diseases impair cognitive functions, from mild cognitive impairment to severe dementia with Alzheimer’s disease being the most prevalent dementia [99]. Some muscle strengthening programs have shown cognitive enhancer effects in these populations [92,93,94,96]. The characteristics of the resistance training program design are described in Table 2. All studies were successful in strengthening muscle [92,94,96]. The Study of Mental and Resistance Training (SMART) program improved cognitive impairments associated with Alzheimer’s disease, as assessed by the ADAS-Cog instrument in community-dwelling adults [93,94]. The resistance training improved declarative memory of elderly women, even after two years of follow-up [96]. Declarative (or explicit) memory dependent on the hippocampus and related cortex are crucial for the memory of facts, events, faces, and environments [100]. However, muscle strengthening did not modify hippocampal atrophy of aged women, but reduced cortical white matter atrophy [96]. 

Preclinical experimental studies focused on the effects of resistance training in the hippocampus of adult male rats. Cassilhas and colleagues [98] demonstrated that resistance training improves spatial memory and serum IGF-1 levels in adult male rats. They also demonstrated an exercise-induced increase in IGF-1R and AKT phosphorylation, as well as in density of IGF-1, synapsin, and synaptophysin in the hippocampus of rats. Neurotrophin TRκB receptor levels were not modified. Gomes and colleagues [47] showed that resistance training increased neurogenesis and decreased apoptosis signaling in the dentate gyrus of adult male rats, however BDNF levels were not modified in the dentate gyrus and CA3 area of the hippocampus after exercise. Vilela et al., [48] demonstrated that resistance training improved spatial memory and strengthened neurotrophin signaling in the hippocampus, unlike young animals [47,98]. All of these well-designed animal and human studies reinforce the feasibility and cognitive benefits of resistance training on elderly cognition.

Clinical studies have also demonstrated the benefits of strength exercise in the executive function [93,94,95], a set of cognitive processes necessary for behavior control that facilitate the attainment of chosen goals. Cuttler and colleagues [101] demonstrated that a single strength exercise session in healthy youths improved prospective memory (the ability to remember intended actions in the future). The home-based Otago Exercise Program demonstrated that resistance training improved response inhibition by 12.8% in the elderly in fall-dedicated clinics, while control sedentary presented a deterioration of response inhibition [95,102], which is important for self-control and resistance to temptations and impulsiveness [103].

**Figure 3:**
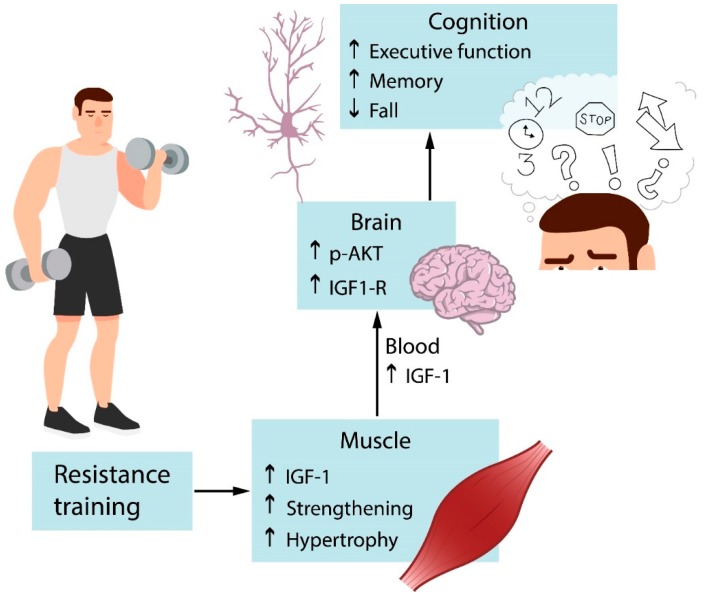
Effects of resistance exercise on cognition. Resistance training activates IGF-1 signaling in muscles, increasing muscle mass and strength. IGF-1 produced by muscles reaches the brain via circulation and binds to specific receptors that lead to the activation of signaling pathways. It acts on specific targets and results in improved cognition. IGF-1 = Insulin growth factor.

Cognitive inhibition is strongly associated with working memory. The core executive function of working memory involves storing information and mentally working with it [96,103]. Aging declines speed of processing, working memory, inhibitory function, and long-term memory, as well as decreases brain structure size and white matter integrity [104]. The home-based Strong for Life program improved verbal working memory of older adults with at least one disability [92]. However, these cognitive benefits were observed in the elderly who reached moderate-high resistance training intensities and not in the elderly who performed low-intensity exercise. Working memory supports inhibitory control and temporarily stored information is crucial for goal behavior with inhibition of errors. The opposite is also true. Inhibitory control supports working memory, goal objective needs focus and resists noise. Impaired working memory during aging is also associated with decreased processing speed, which is the efficiency with which an individual is able to perceive and act upon a stimulus [105]. Yoon et al. [97] demonstrated that resistance training in the elderly without major health problems improved processing speed. The only mechanism reported for the executive cognitive enhancer effects of resistance training in the elderly is the reduction of cortical white matter atrophy, as previously described [96]. Figure 3 depicts that resistance training improves serum IGF-1 levels and hippocampal IGF-1 signaling. Muscle strengthening also boosts cognitive and executive functions.

## 5. Conclusions

The effects of resistance exercise on the brain are not yet fully understood due to the few studies available so far. Although promising, these studies do not allow for a definitive conclusion regarding the effects resistance exercise on the brain’s redox and cognitive mechanisms. In some cases, associations were speculative and further investigations are required. In this regard, future studies in animals and humans may fill these gaps and contribute to further understanding the effects of resistance exercise on the brain.

## Figures and Tables

**Table 1:** Recent pre-clinical (part A) and clinical (part B) studies related to the effects of resistance (or strength) training on the brain.

	**Part A-Pre-Clinical Studies**			
	**Aim**	**Species**	**Results**	**Year of Publication and Reference**
1	Investigate the influence of aerobic and resistance training on the Central Nervous System in an experimental animal model of multiple sclerosis.	Mouse	Although aerobic exercise showed more prominent effects, strength exercise also contributed to neuroprotective mechanisms by modulating inflammatory parameters and oxidative stress.	2017, [7]
2	Investigate the effects of strength and aerobic training on mitochondrial and inflammatory parameters in an experimental animal model of Parkinson’s disease.	Mouse	Both training protocols induced neuroprotection by modulating mitochondrial function and cerebral inflammation parameters.	2015, [8]
3	Investigate the effects of two types of physical training on depressive-like behavior, and levels of proBDNF, brain-derived neurotrophic factor (BDNF), TrkB, in a mouse model of Parkinson’s disease.	Rat	Both types of physical exercise prevented depressive-like behavior and restored levels of proBDNF, BDNF, and TrkB in the striatum and hippocampus.	2014, [9]
4	Investigate the effects of the nandrolone decanoate during a strength exercise program on cell proliferation, apoptotic status, and BDNF expression in the rat hippocampus.	Rat	The increase in the immunoreactivity of anti-apoptotic protein Bcl-2 (DG and CA3) induced by strength exercise was diminished by nandrolone decanoate.	2014, [47]
5	Investigate the effect of aerobic and resistance training on spatial memory and hippocampal plasticity in aging rats.	Rat	Both aerobic and strength training improved spatial memory by distinct molecular neuroplastic mechanisms.	2017, [48]
6	Verify the effects of resistance exercise on memory and motor co-ordination in male and female rats treated with monosodium glutamate.	Rat	Resistance exercise reduced memory and motor co-ordination impairment caused by monosodium glutamate.	2017, [67]
7	Investigate the effects of aerobic, resistance, and combined exercise on Alzheimer’s disease animal model.	Rat	All training models reduced disease oxidative stress scores, increased antioxidant activity, and improved brain plasticity.	2017, [68]
8	Investigate the effects of resistance exercise on the number of seizures, long-term memory, and expression of signaling proteins in rats with epilepsy.	Rat	Resistance exercise reduced memory deficits in rats with epilepsy and increased Insulin-like growth factor 1 and BDNF levels, as well as signaling protein activation.	2017, [69]
9	Investigate the expression of inflammatory cytokines and chemokines and signaling proteins in aged rats undertaking aerobic and resistance exercise.	Rat	No significant difference in cytokines or signaling proteins in the cortex and hippocampus of old rats in response to resistance training was seen.	2018, [70]
10	Verify the effects of resistance exercise training on hypothalamic glucagon-like peptide 1 receptor (GLP-1R) levels and its related signaling mechanisms in type II diabetes (T2DM).	Rat	Resistance training increased GLP-1R mRNA, protein kinase A, glucose transporter 2, and AKT and significantly decreased PKC-iota). Antioxidant enzymes and apoptotic factors were significantly improved in the hypothalamus.	2019, [71]
11	Investigate the effects of aerobic and resistance exercise on the recognition memory and acetylcholinesterase (AChE) activity in a beta-amyloid (Aβ) model of AD in rats.	Rat	Both aerobic and strength training improved the exploration index. AChE activity increased in the Aβ-injected sedentary group but declined in the aerobic and resistance exercise groups.	2019, [72]
	**Part B-Clinical Studies**			
1	Investigate the effects of acute resistance exercise to-fatigue on serum BDNF levels in adult men (serum).	Human	Resistance exercise provided the necessary stimulus to increase peripheral serum BDNF.	2017, [43]
2	Identify the effects of strength training on hippocampus volume in older women.	Human	Hippocampus volume was significantly increased after strength exercise.	2017, [73]
3	Compare full-body versus split-body resistance training on BDNF levels in adult men.	Human	Resistance exercise increased BDNF levels in the serum of adult men.	2018, [74]
4	Compare the response of neurotrophic factors NT3, NT4, and BDNF following one session of high-intensity exercise, resistance training, or both, in physically inactive overweight adult men.	Human	Acute resistance training and combined exercise increased neurotrophic factors in physically inactive overweight adults.	2018, [75]
5	Investigate the effects of aerobic, resistance, and combined training on resting serum BDNF levels in adolescents with overweight and obesity.	Human	All training models increased BDNF levels.	2018, [76]
6	Verify the effects of exercise combined with low- and high-intensity strength exercise in the brain.	Human	Strength exercise weakened aerobic exercise-induced cognitive improvements and hippocampal neurogenesis.	2018, [77]

Systematic database searches of PubMed, Web of Science, Scopus, and Medline were performed to identify peer-reviewed studies from the 2000s. Combinations of keywords related to brain, disease, aerobic/resistance, or strength physical exercise were used.

**Table 2:** Resistance training for cognitive enhancer effects in the elderly.

Program	RCT	Outcome	Resistance Training (RT)				Year of Publication and Reference
			Duration	Volume	Overload	Supervision	
Otago exercise program	Yes	Prevent fall	6 mo 3 times/wk	2 × 10 repetitions	Ankle cuffs	No	2008, [95]
Strong for Life	Yes	Muscle strength	6 mo 3 times/wk 25 min	Uninformed	Elastic bands	No	2006, [92]
Muscle strengthening	Yes	Muscle strength	52 wk 1–2 times/wk 40 min	2 × 6–8 repetitions 7RM method	Pneumatic Free weights (dumbbells)	Yes	2015, [96]
Study of Mental and Resistance Training (SMART)	Yes	Cognition	6 mo 2 times/wk	3 × 8 repetitions 80% 1RM 15–18 Borg scale	Pneumatic Free weights (dumbbells)	Yes	2011, 2017, [93,94]
Muscle strengthening	No	Cognition	16 wk 3 times/week 40 min	2–3 × 12–15 repetitions	Elastic bands High speed	Yes	2018, [97]

References are the search results PubMed and Scopus databases in 2019, February/March. Keywords: Clinical studies, muscle strength, resistance training, cognition, memory, and executive function.
